# Physical activity levels and energy intake according to the presence of metabolic syndrome among single-household elderly in Korea: Korean National Health and Nutrition Examination Survey 2016–2018

**DOI:** 10.3389/fpubh.2023.1063739

**Published:** 2023-03-02

**Authors:** Eun-Sook Sung, Jonghoon Park

**Affiliations:** Exercise Nutrition and Biochemistry Laboratory, Department of Physical Education, Korea University, Seoul, Republic of Korea

**Keywords:** energy intake, metabolic syndrome, physical activity, single household, elderly

## Abstract

**Background:**

Exercise and dietary and nutritional intake affect the risk and prevalence of metabolic syndrome (MetS) in elderly people, effects that may differ according to sex in elderly single households (ESH). This study aimed to analyze the differences in physical activity (PA) levels and energy intake according to sex and prevalence of MetS among elderly people in Korea to investigate the relationships between these factors.

**Methods:**

Data from 893 elderly individuals (aged >65 years) were obtained from the Korean National Health and Nutrition Examination Survey (2016–2018). We analyzed PA levels (occupational and recreational PA and place movement) and energy intake (EI; total, carbohydrate, protein, and fat), and found that there were sex differences in both according to the presence or absence of MetS in ESH.

**Results:**

Among both males and females, the MetS group had a significantly lower recreational moderate PA than the non-MetS group. However, total PA in males was significantly higher in the non-MetS than in the MetS group, but there was no significant difference in females. Furthermore, the EI of females did not differ in the presence or absence of MetS, except for fat intake, and in the nutritional intake of ESH in males, no difference was found in the presence or absence of MetS. We also found that in Odd ratio, “active” was associated with lowering high waist circumference (OR = 0.40, 95%CI = 0.21–0.76), and “very active” was associated with lower MetS occurrence (OR = 0.51, 95%CI = 0.33–0.81) and low high-density lipoprotein cholesterol (HDL-C) (OR = 0.55, 95%CI = 0.37–0.83).

**Conclusions:**

Therefore, in the MetS group of ESH, there was a significant correlation of the MetS component in PA rather than EI. Male ESH require interventions that increase PA, while female ESH require nutrition interventions that increase and balance PA. Therefore, a new program is needed that promotes continuous interest and healthy lifestyles in consideration of the characteristics of ESH.

## 1. Introduction

The proportion of the elderly population in Korea is increasing and will reach 20.3% by 2025, and it is expected that about half of all households will be elderly by 2047 (1). In addition, due to the low fertility rate and aging population, a typical household consisting of a man, a woman, and one or two children is gradually shifting to a one-person household. According to the National Statistical Office, the proportion of single households among the elderly aged 60 years and over is expected to increase from 30% in 2015 to 54% in 2045 (2).

The most common cause of medical expenses among the elderly is metabolic syndrome (MetS), which causes chronic diseases, such as cardiovascular disease and diabetes (3). According to the Korean National Health and Nutrition Survey (KNHANES), the incidence of MetS has increased by 0.6% every 10 years since 1998 (4). MetS comprises a bundle of metabolic risk factors, including hyperglycemia, dyslipidemia, abdominal obesity, and hypertension (5). Higher levels of physical activity (PA) may protect against the progression of cardiovascular disease (6), type 2 diabetes (7), and MetS (8–10), and people with MetS who participate in PA have a lower risk of mortality from MetS than those with normal weight; however, sedentary adults have been reported as evidence of a high risk of mortality from MetS (11). Moreover, the consumption of a Western diet, meat and fried foods favors the occurrence of MetS (12). Although the main aim of nutrition is the prevention and treatment of nutritional deficiencies, overnutrition can negatively affect health and cause many metabolic disorders such as diabetes, obesity, hypertension, and hyperlipidemia (13–17). A previous study showed that all five components of MetS were improved through modifications in energy intake (EI) and exercise (18). Therefore, EI, similar to exercise, is an important component of MetS prevention. A previous study in Korea found that adult single households were associated with insufficient PA and unbalanced EI habits compared to mixed households (19). A decrease in moderate PA and walking was induced at low PA levels, and reduced PA contributed to the incidence of MetS in elderly single households (ESH) (20). Among elderly people, living alone has been found to be associated with specific health concerns, such as poor EI and PA (21). A previous study demonstrated that elderly people, who eat alone was associated with several health problems, including MetS (22), lower caloric intake, and a less-varied diet (23). Elderly people who lived alone had a higher risk of MetS than those who did not (24).

The importance of sex has also been considered when distinguishing the influence of living alone on MetS (25). Elderly male living alone are associated with poorer diets (26), whereas elderly female living alone are more likely to have physical limitations (27). A previous study reported that elderly female living alone were not socially isolated nor were they at high risk of deterioration in their functional health status. Instead, they reported more contact with close friends outside the household (28). A previous study reported that elderly female living alone did not have a high risk of deterioration in their functional health status and that they engaged in more activities with friends outside the household (29). These sex differences in the characteristics of elderly people living alone could be relevant by PA, and EI differences in the relationship between living alone and MetS. However, there is little research on housing and MetS among elderly people, particularly among ESH with sex differences.

Therefore, this study aimed to analyze the relationship between PA levels, and EI in the presence and absence of MetS, and the sex differences between PA levels, and EI in the presence and absence of MetS in an ESH Korean population, based on data from the 7th Korea National Health and Nutrition Examination Survey (2016–2018), to investigate the relationships between these factors.

## 2. Materials and methods

### 2.1. Sample and design

This study used cross-sectional data from the Korea National Health and Nutrition Examination Survey (KNHANES) conducted by the Korea Centers for Disease Control and Prevention (KCDC) from 2016 to 2018. These data are updated every 3 years. Therefore, we used current data. The details of the study design and data source profiles followed the methods outlined in the guidelines for the use of raw KNHANES data and in the final report on the sampling frame (30).

**Figure 1:**
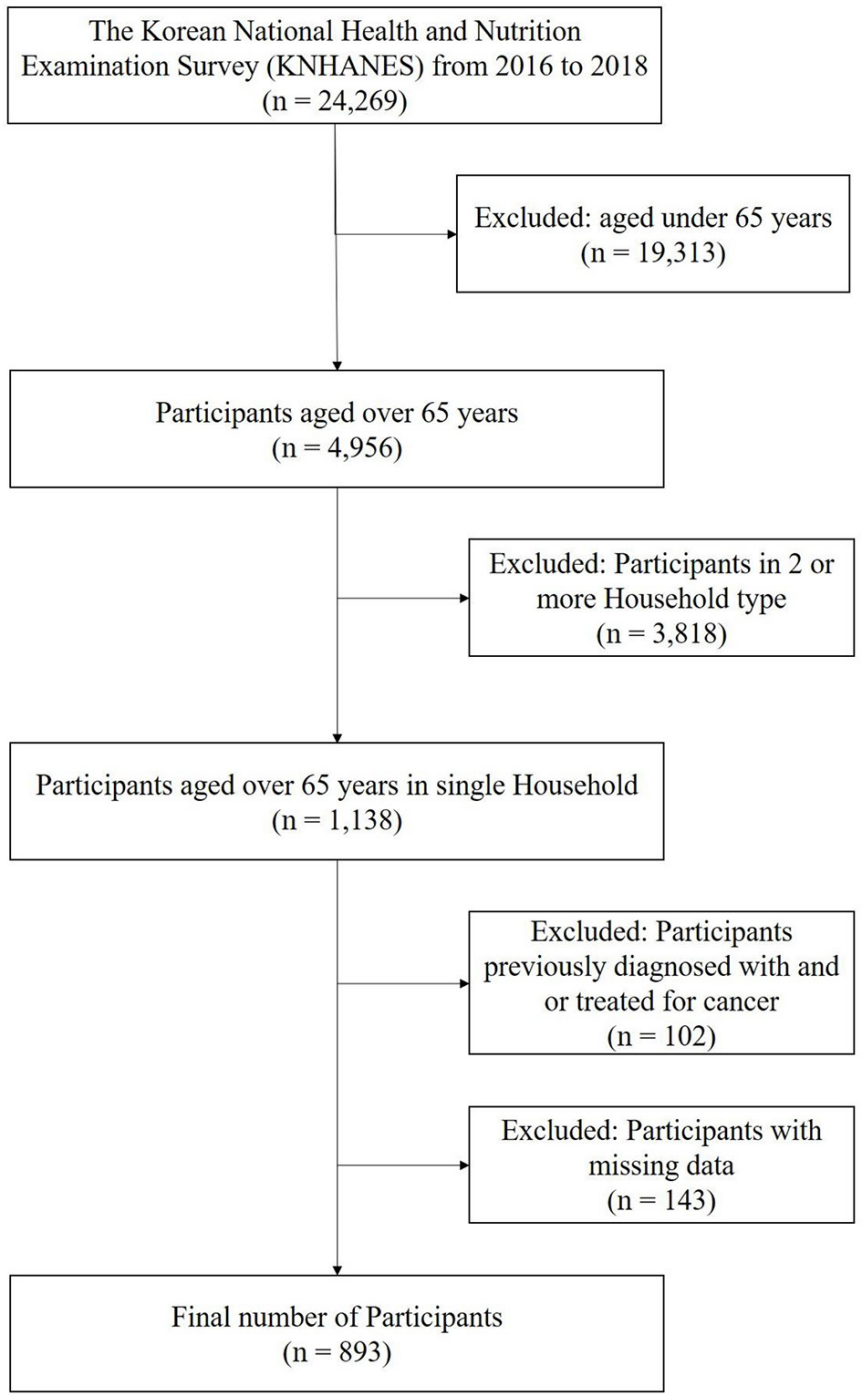
Flow diagram for the selection of study participants.

From 2016 to 2018, 24,269 individuals completed a health interview survey, nutrition surveys, and health examinations, which were conducted according to the Declaration of Helsinki. This is a survey to assess the health and nutritional status of South Koreans and is conducted by the Korea Centers for Disease Control and Prevention. The National Health and Nutrition Examination Survey was approved by the Institutional Review Board of the Korea Centers for Disease Control and Prevention (Reference Number 2018-01-03-P-A). Preceding the survey, all participants were informed about the purpose and procedures of the survey and written informed consent was obtained from each participant prior to involvement in the survey. Nineteen thousand three hundred and thirteen participants with under the age of 65 years were excluded and 4,956 participants over the age of 65 years remained. Moreover, among the 4,965 elderly participants, 3,818 multi-person families were excluded, and 1,138 elderly single-household participants remained. Of these, 893 individuals were included in the study, after excluding 102 persons who had been previously diagnosed with or treated for cancer (gastric, colorectal, liver, cervical, breast, thyroid, lung, and other cancers) and 143 persons who had undergone surgery for other indications (Figure 1). In total, 893 ESH (age between 65 and 80 year) were included in this study and on the day of the survey, the participants participated in the survey without taking medications for chronic diseases (ex. high blood pressure, hyperlipidemia, diabetes).

### 2.2. Measures

Table 1 shows the characteristics of the participants according to sex. The presence or absence of MetS was assessed through measurement of waist circumference, blood pressure, and fasting blood glucose, triglyceride (TG), and high-density lipoprotein cholesterol (HDL-C) levels. These MetS measures were determined using a survey. The KHANES data presented PA variables of occupational activity, recreational activity, and transport in metabolic equivalent (MET)-minutes/week based on the Global Physical Activity Questionnaire (GPAQ). In addition, the KHANES data included dietary outcomes on consumption levels using the 24-h recall method by interviewing target elderly single households in person.

**Table 1:** Descriptive characteristics of the participants.

	**Total (*****n*** = **893)**			**Male (*****n*** = **220)**			**Female (*****n*** = **673)**		
	**Non-MetS (*****n*** = **626)**	**MetS (*****n*** = **267)**	***p*** **-value**	**Non-MetS (*****n*** = **145)**	**MetS (*****n*** = **75)**	***p*** **-value**	**Non-MetS (*****n*** = **481)**	**MetS (*****n*** = **192)**	***p*** **-value**
Age (years)	74.3 ± 0.2	74.7 ± 0.3	0.931	72.7 ± 0.4	72.9 ± 0.8	0.903	74.8 ± 0.2	75.4 ± 0.4	0.039^*^
Height (cm)	154.2 ± 0.4	155.1 ± 0.6	0.348	165.2 ± 0.5	165.6 ± 0.7	0.997	150.8 ± 0.3	150.9 ± 0.5	0.507
Body weight (kg)	56.2 ± 0.4	62.9 ± 0.7	0.133	61.7 ± 0.9	70.1 ± 1.0	0.860	54.6 ± 0.4	60.1 ± 0.7	0.485
BMI (kg/m^2^)	23.6 ± 0.1	26.1 ± 0.2	0.335	22.6 ± 0.2	25.5 ± 0.3	0.975	23.9 ± 0.2	26.4 ± 0.3	0.449
Alcohol (%)	30.6 ± 2.1	28.7 ± 3.1	0.157	65.1 ± 4.0	58.1 ± 7.7	0.568	20.2 ± 2.1	17.0 ± 2.8	0.155
Smoking (%)	8.7 ± 1.2	12.1 ± 2.7	0.099	32.7 ± 3.5	29.7 ± 7.5	0.047^*^	1.4 ± 057	5.1 ± 1.6	0.021^*^

Values are expressed as mean ± standard error; BMI, body mass index; alcohol, percentage of drinking at least once a month in the past year; smoking, percentage of currently smoking; MetS, metabolic syndrome.

* p < 0.05.

### 2.3. Metabolic syndrome

The diagnosis of MetS was determined the new harmonized guidelines of the National Cholesterol Education Program-Adult Treatment Panel III (31) and the American Heart Association and the National Heart Lung and Blood Institute (32). For waist circumference, we followed the criteria suggested by the Korean Society for the Study of Obesity (33). MetS was diagnosed if participants had three or more of the following (34): waist circumference >90 cm (male) or >85 cm (female), systolic blood pressure (SBP) ≥130 mmHg or diastolic blood pressure (DBP) ≥85 mmHg, fasting TG levels ≥150 mg/dL, fasting HDL-C levels < 40 mg/dL (male) or < 50 mg/dL (female), and fasting glucose (FG) levels ≥110 mg/dL.

### 2.4. Physical activity

The GPAQ comprises 16 questions grouped to capture PA in different behavioral domains: work, transport, and recreational activities. Five domains of PA were analyzed: vigorous-intensity work, moderate-intensity work, transport, vigorous-intensity recreation, and moderate-intensity recreation. Participants answered the five domains freely, without any additional options regarding how many times a week and how many minutes per day they performed the activity. The World Health Organization (WHO) GPAQ analysis guidelines were used to analyze the GPAQ data (35). We estimated that a person's caloric expenditure was four times higher when they were moderately active and eight times higher when they were vigorously active compared to sitting quietly. Therefore, when calculating the total energy expenditure of an individual using GPAQ data, four METs were assigned to the time spent in moderate activity and eight METs were assigned to the time spent in vigorous activity, and the details are as follows:

- Vigorous intensity activity: occupational (MET) = 8.0 × vigorous intensity physical activity (day/week) × 1-day vigorous intensity physical activity (minutes/day)
- Moderate intensity activity: occupational (MET) = 4.0 × moderate intensity physical activity (day/week) × 1-day moderate intensity physical activity (minutes/day)
- Vigorous intensity activity: recreational (MET) = 8.0 × vigorous intensity physical activity (day/week) × 1-day vigorous intensity physical activity (minutes/day)
- Moderate intensity activity: recreational (MET) = 4.0 × moderate intensity physical activity (day/week) × 1-day moderate intensity physical activity (minutes/day)
- Place movement (MET) = 4.0 × place movement physical activity (day/week) × 1-day place movement physical activity
- Total Physical Activity (MET) = vigorous intensity activity: occupational + moderate intensity activity: occupational + vigorous intensity activity: recreational + moderate intensity activity: recreational + place movement.

PA levels were divided into four groups: inactive (0–249 MET min/week), somewhat active (250–499 MET min/week), active (500–999 MET min/week), and very active (>1,000 MET min/week). These thresholds are based on their equivalence to the following PA thresholds: 250 MET min/week corresponds to an energy expenditure dose equal to half the threshold, 500 MET min/week corresponds to the minimum threshold, and 1,000 MET min/week corresponds to twice the minimum threshold (36).

### 2.5. Energy intake

Dietary outcomes were obtained using the 24-h recall method by interviewing target households in person. Nutrition survey data were collected from participants' homes by trained dietitians 1 week after the health interview and health examination. The daily energy intake was calculated using the Korean Food and Nutrient Database of the Rural Development Authority. The following items were included in the analyses: total energy intake, carbohydrate intake, protein intake, and daily fat intake. Energy intake data were converted into kcal using the conversion factor of 4 kcal/g for carbohydrates and proteins and 9 kcal/g for fats. Energy intake was categorized by dividing the ratio by the estimated energy requirement (EER). The EER is the average dietary energy intake predicted to maintain energy balance in healthy, normal-weight individuals of a given age, sex, weight, height, and level of physical activity in good health. We used individual EER based on the Institute of Medicine (IOM) equations, based on body mass index (BMI), age, and sex. We then selected physical activity levels (PAL) to estimate the energy requirements. Values < 20% EER (< 0.8) were considered as lower intake, whereas values above 1.2 as higher intake (37, 38).

### 2.6. Statistical analysis

Continuous variables are presented as means and standard errors. The normality of the distribution of all outcome variables was tested with the Kolmogorov–Smirnov test. Post-mortem independent *t*-test was performed to analyze risk factors for MetS, as well as PA levels and energy intake between the non-MetS and MetS groups and sex characteristics of the dependent variable in each group. One-way analysis of variance (ANOVA) was used to analyze the differences in risk factors for MetS, PA levels, and energy intake between participants with and without MetS, and between males and females. The relationship between PA levels or energy intake and MetS was also determined using logistic regression analysis after controlling for covariates, which included gender, smoking, alcohol consumption, and body mass index. The results of the logistic regression analysis are presented in the form of odds ratios (ORs) and their associated 95% confidence intervals (CIs). Statistical analyses were performed using SPSS version 25.0 for Windows (IBM Corp., Armonk, NY, USA). The level of significance was set at *p* < 0.05.

## 3. Results

Table 2 shows the differences in variables considered risk factors for MetS according to the presence or absence of MetS and sex.

**Table 2:** Total and sex differences in metabolic syndrome components.

**Variables**	**Total**		**Male**		**Female**	
	**Non-MetS (*n* = 626)**	**MetS (*n* = 267)**	**Non-MetS (*n* = 140)**	**MetS (*n* = 63)**	**Non-MetS (*n* = 366)**	**MetS (*n* = 275)**
Waist circumference (cm)	82.6 ± 0.4	90.9 ± 0.5	83.2 ± 0.8	92.3 ± 0.8	82.4 ± 0.4	90.4 ± 0.7
TG (mg/dL)	108.6 ± 2.3	188.5 ± 10.4	102.8 ± 4.1	176.9 ± 13.5	110.3 ± 2.5	193.1 ± 13.1
HDL-C (mg/dL)	51.5 ± 0.4	40.8 ± 0.7	50.9 ± 1.2	39.5 ± 1.3	51.7 ± 0.5	41.3 ± 0.8
SBP (mmHg)	127.2 ± 0.8	137.0 ± 1.2	124.2 ± 1.2	130.2 ± 1.9	128.1 ± 0.9	139.7 ± 1.4
DBP (mmHg)	71.3 ± 0.5	73.6 ± 0.7	70.9 ± 0.8	72.4 ± 1.2	71.4 ± 0.5	74.0 ± 0.7
FG (mg/dL)	104.7 ± 1.1	123.5 ± 2.9	107.8 ± 2.9	123.0 ± 6.2	103.8 ± 1.1	123.7 ± 2.9

Values are expressed as mean ± standard error.

MetS, metabolic syndrome; TG, triglyceride; HDL-C, high-density lipoprotein cholesterol; SBP, systolic blood pressure; DBP, diastolic blood pressure; FG, fasting glucose.

Table 3 shows significant difference in recreational moderate PA between Mets and non-MetS in total (*p* < 0.01), males (*p* < 0.05), and females (*p* < 0.05). There was significant difference in total PA between Mets and non-MetS in total (*p* < 0.05) and males (*p* < 0.05). In females, there was not a significant difference in total PA.

**Table 3:** Levels of physical activity.

**Physical activity (MET × min/week)**	**Group**	**Total (*n* = 893)**	**Male (*n* = 220)**	**Female (*n* = 673)**	**Sex difference (SD)**	
					***t***	***p***
Occupational vigorous	Non-MetS	6.9 ± 5.1	0.0 ± 0.0	9.0 ± 6.7	0.051	0.919
	MetS	1.5 ± 1.4	5.4 ± 5.3	0.0 ± 0.0	0.050	0.966
	***p-value***	0.315	0.307	0.178		
Occupational moderate	Non-MetS	42.7 ± 16.6	65.1 ± 47.1	36.1 ± 18.0	0.065	0.720
	MetS	23.4 ± 8.2	41.1 ± 23.7	16.4 ± 8.1	0.055	0.831
	***p-value***	0.262	0.647	0.300		
Transport	Non-MetS	360.8 ± 33.5	460.0 ± 97.6	331.1 ± 29.5	0.332	0.128
	MetS	308.1 ± 42.7	266.5 ± 70.0	324.6 ± 52.9	0.077	0.631
	***p-value***	0.332	0.105	0.911		
Recreational vigorous	Non-MetS	9.0 ± 4.7	17.6 ± 12.8	6.4 ± 4.9	0.053	0.866
	MetS	11.0 ± 7.8	36.0 ± 27.3	1.1 ± 1.1	0.066	0.712
	***p-value***	0.829	0.545	0.289		
Recreational moderate	Non-MetS	90.4 ± 18.0	171.4 ± 40.2	66.2 ± 20.4	0.626	0.023^*^
	MetS	24.0 ± 9.2	52.3 ± 29.3	12.8 ± 5.4	0.092	0.548
	***p-value***	0.001^**^	0.016^*^	0.012^*^		
Total physical activity	Non-MetS	509.9 ± 44.8	714.1 ± 129.4	448.7 ± 41.3	0.268	0.181
	MetS	368.0 ± 43.8	401.3 ± 76.7	354.9 ± 54.3	0.053	0.869
	***p-value***	0.021^*^	0.036^*^	0.150		

Values are expressed as mean ± standard error.

MetS, metabolic syndrome.

* p < 0.05.

** p < 0.01.

Table 4 shows the differences in energy intake according to the presence or absence of MetS and sex. There was no significant difference in total energy intake, carbohydrate intake, or fat intake between the MetS and non-MetS groups in the total group and males. In females, fat intake (*p* < 0.05) was lower in those with MetS than in those without MetS. As shown by sex differences, females had significantly lower total energy intake (*p* < 0.001), carbohydrate intake (*p* < 0.001), fat intake (*p* < 0.05), and protein intake (*p* < 0.001) in the non-MetS group. In MetS, females had significantly lower total energy intake (*p* < 0.001), carbohydrate intake (*p* < 0.001), and protein intake (*p* < 0.01) but tended to have lower fat intake than males (*p* = 0.053).

**Table 4:** Energy intake.

**Variables**	**Group**	**Total (*n* = 893)**	**Male (*n* = 220)**	**Female (*n* = 673)**	**SD**	
					***t***	***p***
Total energy intake (kcal)	Non-Mets	1,550.6 ± 29.9	1,953.3 ± 63.9	1,405.9 ± 28.5	0.998	0.000^***^
	Mets	1,537.3 ± 63.7	1,949.9 ± 147.8	1,351.2 ± 53.5	0.954	0.000^***^
	***p***	0.854	0.983	0.338		
Carbohydrate intake (%)	Non-Mets	69.21 ± 1.32	64.55 ± 1.90	71.53 ± 1.54	0.987	0.000^***^
	Mets	70.15 ± 2.35	65.56 ± 4.02	73.09 ± 3.05	0.899	0.001^**^
	***p***	0.916	0.842	0.688		
Fat intake (%)	Non-Mets	15.09 ± 0.58	15.71 ± 1.29	14.79 ± 0.58	0.724	0.011^*^
	Mets	13.35 ± 1.00	14.40 ± 1.94	12.66 ± 0.87	0.489	0.053
	***p***	0.096	0.567	0.011^*^		
Protein intake (%)	Non-Mets	12.98 ± 0.34	13.37 ± 0.68	12.78 ± 0.34	0.985	0.000^***^
	Mets	12.91 ± 0.68	13.23 ± 1.23	12.73 ± 0.68	0.842	0.003^**^
	***p***	0.819	0.900	0.420		

Values are expressed as mean ± standard error.

MetS, metabolic syndrome; SD, sex differences.

* p < 0.05.

** p < 0.01.

*** p < 0.001.

Tables 5, 6 show the average values of PA levels and energy intake factors. More than 50% of the total ESH (*n* = 536) were “inactive” (0–249 MET min/week), both males (*n* = 123) and females (*n* = 413) showed also that more than 50% of the ESH were “inactive”, and those who were “very active” (>1,000 MET min/week) were found to be the least (*n* = 106). Most ESH (*n* = 802) were found to have low energy intake (energy intake/EER < 0.8), both males (*n* = 183) and females (*n* = 619) showed the same tendency, and those with high energy intake (energy intake/EER > 1.2) were less (*n* = 31).

**Table 5:** Classification of physical activity levels.

**Physical activity level**	**MET min/week (mean** ±**SE)**		
	**Total (*n*)**	**Male (*n*)**	**Female (*n*)**
Inactive (0–249 MET min/week)	29.7 ± 3.4 (536)	24.8 ± 6.2 (123)	31.2 ± 3.9 (413)
Somewhat active (250–499 MET min/week)	441.9 ± 8.7 (120)	406.2 ± 14.7 (26)	453.2 ± 9.9 (94)
Active (500–999 MET min/week)	837.7 ± 13.8 (131)	838.6 ± 32.2 (30)	837.5 ± 15.3 (30)
Very active (>1,000 MET min/week)	2,245.1 ± 144.3 (106)	2,223.1 ± 213.6 (41)	2,260.0 ± 208.5 (65)

Values are expressed as mean ± standard error.

MET, metabolic equivalent of task.

**Table 6:** Classification of energy intake.

**Energy intake factors**	**Energy intake/EER (mean** ±**SE)**		
	**Total** ***(n)***	**Male** ***(n)***	**Female** ***(n)***
Lower energy intake (energy intake/EER < 0.8)	0.28 ± 0.01 (802)	0.31 ± 0.01 (183)	0.27 ± 0.01 (619)
Moderate energy intake (0.8 ≤ energy intake/EER ≤ 1.2)	0.94 ± 0.01 (60)	0.93 ± 0.02 (60)	0.94 ± 0.02 (38)
Higher energy intake (energy intake/EER > 1.2)	1.52 ± 0.05 1.53 (31)	1.43 ± 0.05 (15)	1.65 ± 0.08 (16)

Values are expressed as mean ± standard error.

EER, estimated energy requirements.

The ORs for MetS and MetS components according to PA level and energy intake are presented in Table 7 and was adjusted for sex, smoking, alcohol consumption, and age. We found that “active” was associated with lowering high waist circumference (OR = 0.40, 95%CI = 0.21–0.76, *p* < 0.01), and “very active” was associated with lower MetS occurrence (OR = 0.51, 95%CI = 0.33–0.81, *p* < 0.01) and low HDL-C (OR = 0.55, 95%CI = 0.37–0.83, *p* < 0.01).

**Table 7:** Odds ratio (95% CI) for MetS and MetS components according to physical activity levels and energy intake.

**Factors**	**MetS**	**Large waist circumference**	**High triglycerides**	**Low HDL-C**	**High blood pressure**	**High glucose**
**Physical activity factors**						
Inactive (*n* = 536)	1.00 (reference)	1.00 (reference)	1.00 (reference)	1.00 (reference)	1.00 (reference)	1.00 (reference)
Somewhat active (*n* = 120)	0.66 (0.39–1.13)	0.91 (0.45–1.82)	0.58 (0.32–1.04)	0.77 (0.45–1.33)	0.83 (0.53–1.28)	0.97 (0.60–1.56)
Active (*n* = 131)	0.59 (0.34–1.04)	**0.40 (0.21–0.76)** ^*^	0.60 (0.36–1.00)	0.98 (0.63–1.51)	0.78 (0.49–1.22)	0.80 (0.51–1.24)
Very active (*n* = 166)	**0.51 (0.33–0.81)** ^*^	0.74 (0.41–1.34)	0.64 (0.41–1.00)	**0.55 (0.37–0.83)** ^*^	0.98 (0.67–1.43)	0.81 (0.50–1.32)
**Energy intake factors**						
Lower energy intake (energy intake/EER < 0.8)	1.00 (reference)	1.00 (reference)	1.00 (reference)	1.00 (reference)	1.00 (reference)	1.00 (reference)
Moderate energy intake (0.8 ≤ energy intake/EER ≤ 1.2)	0.58 (0.32–1.08)	0.72 (0.31–1.64)	0.61 (0.33–1.14)	0.57 (0.32–1.01)	0.66 (0.35–1.26)	1.25 (0.70–2.24)
Higher energy intake (energy intake/EER > 1.2)	0.74 (0.33–1.68)	1.33 (0.44–4.03)	0.87 (0.35–2.17)	0.81 (0.35–1.88)	1.16 (0.56–2.41)	1.13 (0.51–2.47)

Data are presented as odds ratios [95% confidence intervals (CIs)] and was adjusted for sex, smoking, alcohol consumption, and age.

OR, odds ratio; MetS, metabolic syndrome; HDL-C, high-density lipoprotein cholesterol; EER, estimated energy requirement.

^*^p < 0.01 vs. reference.

## 4. Discussion

This study examined the differences in PA levels and EI according to the presence or absence of MetS and sex among ESH in Korea to understand the correlations between these factors. The total MetS group engaged in significantly less recreational moderate PA and total PA than the non-MetS group. In particular, the recreational moderate and total PA levels were significantly lower in males with MetS, whereas females with MetS had only significantly lower recreational moderate PA. “Active” was associated with lowering high waist circumference and “Very active” with lower MetS occurrence and low HDL-C as using adjustments for sex, smoking, alcohol consumption, and age. Examination of differences in energy intake according to the presence or absence of MetS showed that there was a significant difference only in fat intake in females, which was lower in those with MetS; no significant difference was found in the total group or in males. According to the odds ratio of EI, “moderate energy intake” was found to be associated with only HDL-C, and there was no association between components of EI and MetS. Taken together, our results suggest that ESH in MetS component is more strongly associated with PA than EI.

In the present study, we found that higher recreational moderate PA was associated with low MetS morbidity in both males and females. A previous study of 477 people (aged 55–80 years) in Spain found that the MetS group had lower energy expenditure and less leisure-time PA (< 4 MET) than the non-MetS group (39). Jung et al. (40) investigated 3,720 participants in the Korea National Health and Nutrition Examination Survey (KNHANES) from 2016 to 2018, aged >65 years irrespective of household type, and compared MetS risk with PA level. They reported that the extent of PA according to the presence or absence of MetS differed more in terms of recreational PA than occupational PA. Our study also showed that MetS was more inactive than non-MetS in the total group; therefore, it appears that recreational moderate PA is significant correlation for MetS in elderly people. In addition, in relation to PA level, Smith et al. (41) and Sarkar et al. (42) reported that having someone (e.g., family and friends) is positively correlated with PA level, whereas living alone may promote a decrease in total PA, which may be as a risk factor for increased occurrence of MetS. Comparing our study with ESH and with mixed households (single and mixed-household type) (40), PA at the total PA and recreational moderate levels of elderly people with MetS in the mixed-household type was higher than in our study; therefore, it can be observed that PA among ESH is low. Moreover, when the total PA level of mixed-households and ESH in our study was compared by sex, there was no significant difference in the presence or absence of MetS in males by mixed-household type, but there was a significant difference in females. Conversely, in our study, there was a significant difference in the presence or absence of MetS in males, but not in females. Previous studies have shown that older female living alone engage in more activities and contact with friends through social relationships than older male living alone (43). There appeared to be no difference in PA levels between the MetS and non-MetS groups due to the social characteristics of female. Furthermore, in the study by Jung et al. (40), according to sex, the male total PA in the MetS group of mixed households was higher and female total PA and recreational moderate PA in the MetS group of mixed households was higher than in our study. This difference can be interpreted as a result according to the type of household, and it can be appears that single households are more exposed to component of MetS because the PA level is lower than that of mixed households.

In our study, an analysis of dietary intake in relation to the presence or absence of MetS was also performed in elderly people. This showed that total EI and carbohydrate, fat, and protein intakes did not show significantly different between MetS and non-MetS in both sexes, with the exception of fat intake in females. According to the results for a mixed household (40), carbohydrate, fat, and protein intakes were also not significantly different between MetS and non-MetS groups in males. However, in females, the total energy intake (carbohydrate, fat, and protein) was significantly different between the MetS and non-MetS groups. This result is supported by a previous study, which reported that the dietary quality and food diversity of females was better than that of males (44). Moreover, the overall low nutrient intake and low nutrient density of meals were the major nutritional problems in the group of ESH. As in the previous study mentioned above, ESH also consumed less than the recommended dietary intake. Therefore, malnutrition rather than a nutrient excess appears to be the problem in ESH, and consumption of a balanced diet may be more important than deficient intake of a single nutrient. These dietary intake patterns of the elderly have been described in more detail in previous studies. Giezenaar et al. reported that low EI is a strong predictor of poor outcomes, including the development of pathological undernutrition and sarcopenia, as well as reduced functioning and frailty; this low EI in the elderly affects the decline in PA (45). Therefore, ESH appear to experience a greater reduction in PA compared with mixed households. Previous research has shown that overconsumption (an unbalanced diet) and reduced physical activity are associated with a variety of problems, including increased insulin resistance, neurohormonal activation, and chronic inflammation (46). These problems are known to be strongly associated with an increased risk of developing atherosclerotic cardiovascular disease (CVD) and MetS (47). Previous research has shown that increasing physical activity and eating a balanced diet are necessary to address these issues (48, 49). Our findings also suggest that a balanced diet and changes in energy expenditure are essential for reducing the prevalence of MetS, and notably, the need for a balanced diet, especially in ESH, has been found to be due to low energy intake rather than high energy intake.

The results of this study should be interpreted with the following limitations in mind. First, we assessed ESH with MetS but did not consider the timing of MetS development or the duration of MetS. Second, PA levels were not determined using heart rate measurements or an accelerometer but were based on survey results, which are prone to errors. Third, this study found only simple differences without establishing causality of underlying the association between PA and nutrition. Finally, the data obtained using the 24-h reminder may not reflect long-term dietary habits. The 24-h recall is essentially a retrospective method of diet assessment, in which an individual is asked about their food and beverage consumption during the previous day or the 24 h. However, a single 24 h-recall may not be representative of the habitual diet at an individual level. The strength of this study is that it analyzed PA levels and energy intake in ESH according to the prevalence of MetS. Most studies examining the relationship between MetS, PA levels, and energy intake have not considered household types in the elderly. In particular, this study classified them according to sex and investigated the PA levels and energy intake of ESH. Therefore, it appears that the risk of MetS depends on lifestyle habits, such as PA level, a balanced dietary and healthy energy intake. In this study, we identified sex-specific aspects of PA levels and energy intake with and without MetS in ESH.

## 5. Conclusions

In this study, we found that there was a sex difference in PA level and EI according to the presence or absence of MetS in ESH. Among both male and female, the MetS group engaged in significantly lower recreational moderate PA than the non-MetS group. However, total PA in males was significantly higher in non-MetS than in MetS, but there was no significant difference in females. Furthermore, the EI of females did not differ in the presence or absence of MetS, except for fat intake, and in the nutritional intake of ESH in males, no difference was found in the presence or absence of MetS. Therefore, in the MetS group of ESH, there was a significant correlation of the MetS component in PA rather than EI. These findings highlight the need for different approaches to implementing PA and nutrition strategies depending on sex in ESH. Male ESH require a program that increases the amount of PA, while female ESH require a nutrition program that increases and balances PA. Therefore, a new program is needed that promotes continuous interest and healthy lifestyles in consideration of the characteristics of ESH.

## Data availability statement

The original contributions presented in the study are included in the article/supplementary material, further inquiries can be directed to the corresponding author.

## Ethics statement

The National Health and Nutrition Examination Survey was approved by the Institutional Review Board of the Korea Centers for Disease Control and Prevention (reference number 2018-01-03-P-A) and the patients/participants provided their written informed consent to participate in this study.

## Author contributions

Conception and study design, statistical analysis, investigation, data interpretation, writing-original draft preparation, and writing-review and editing: E-SS and JP. Supervision: JP. All authors have read and approved the final manuscript.
